# Changes in peanut canopy structure and photosynthetic characteristics induced by an arbuscular mycorrhizal fungus in a nutrient-poor environment

**DOI:** 10.1038/s41598-021-94092-w

**Published:** 2021-07-21

**Authors:** Yinli Bi, Huili Zhou

**Affiliations:** 1 State Key Laboratory of Coal Resources and Safe Mining, China University of Mining and Technology (Beijing), Beijing, 100083 China; 2 College of Geology and Environment, Xi’an University of Science and Technology, Xi’an, 710054 China

**Keywords:** Biological techniques, Biotechnology, Microbiology, Plant sciences

## Abstract

A well-developed canopy structure can increase the biomass accumulation and yield of crops. Peanut seeds were sown in a soil inoculated with an arbuscular mycorrhizal fungus (AMF) and uninoculated controls were also sown. Canopy structure was monitored using a 3-D laser scanner and photosynthetic characteristics with an LI-6400 XT photosynthesis system after 30, 45 and 70 days of growth to explore the effects of the AMF on growth, canopy structure and photosynthetic characteristics and yield. The AMF colonized the roots and AMF inoculation significantly increased the height, canopy width and total leaf area of the host plants and improved canopy structure. AMF reduced the tiller angle of the upper and middle canopy layers, increased that of the lower layer, reduced the leaf inclination of the upper, middle and lower layers, and increased the average leaf area and leaf area index after 45 days of growth, producing a well-developed and hierarchical canopy. Moreover, AMF inoculation increased the net photosynthetic rate in the upper, middle and lower layers. Plant height, canopy width, and total leaf area were positively correlated with net photosynthetic rate, and the inclination angle and tiller angle of the upper leaves were negatively correlated with net photosynthetic rate. Overall, the results demonstrate the effects of AMF inoculation on plant canopy structure and net photosynthetic rate.

## Introduction

Crop canopy structure is a critical factor affecting photosynthesis and commonly refers to the geometry, quantity and spatial distribution of various aboveground parts of the crop. More than 90% of the dry matter accumulated in plants is derived directly or indirectly from photosynthesis and the remainder from nutrients taken up by the roots^1,2^. Canopy structure affects the effective photosynthetic area of the leaves and aspects of the microenvironment such as temperature, humidity and CO_2_ concentration inside the canopy, thereby affecting crop photosynthetic efficiency and yield^3,4^. Canopy structure can be measured directly or indirectly based on leaf area index (LAI) and leaf angle distribution (LAD). Direct measurement is time-consuming, cumbersome and destructive to plants^5^. Using three-dimensional modeling methods to study the characteristics of crop canopy structure can avoid the problems mentioned above, So it gets many scholars's favor. Riczu et al.^6^ established several plant branch models using an on-board laser scanner and compared the columnar branch model established in the Leica system, the 3D mesh tree model established using Geomagic software, and the trunk model using 3D shaping software. They considered the latter two models to be more realistic and more sophisticated. Chang et al.^1^.established a three-dimensional canopy photosynthesis model of rice plants and used it to study the effects of three canopy structural parameters, namely tiller number, tiller angle and leaf angle, on the efficiency of canopy radiation. Xiang et al.^7^ established a non-destructive 3D scanning system using a commercial depth camera and sorghum (*Sorghum bicolor*) as an experimental model to continuously monitor plants of different heights and automatically extract morphological characteristics of sorghum.

Arbuscular mycorrhizal fungi (AMF) are a common group of soil microorganisms. They can form potentially symbiotic relationships with most terrestrial plant species and have the potential to promote plant growth and increase net photosynthetic rate^8–10^. AMF can regulate chloroplast enzyme activity, reduce chlorophyll decomposition rate, accelerate the synthesis of important enzymes required for the chlorophyll peptide chain, promote chlorophyll synthesis, increase chlorophyll content plants^11^, increasing the intensity of photosynthesis^12^, promoting plant growth, increasing leaf area^13^, and then increase the absorption and utilization efficiency of light energy^14,15^, thereby significantly promoting plant biomass^16^.

We used peanut as the experimental material and a three-dimensional laser scanner and an LI-6400 XT photosynthesis system to monitor the dynamic effects of AMF on canopy structure and photosynthetic characteristics to investigate the role of AMF on plant growth, canopy structure, photosynthesis and yield and to estimate plant photosynthetic capacity at later growth stages. The results provide information for improving crop canopy structure and increasing material accumulation and yield through AMF inoculation in nutrient-poor environments.

## Results

### Mycorrhizal colonization rate

After 70 days, we calculated the root colonization rate of each plant, including hyphae, arbuscules, vesicles of AMF, calculated the average colonization rate of the same treatment, and calculated the variation with standard error, and found that the percentage of peanut root length colonized by the AMF was 95.4 ± 1.1(%). No AMF colonization was found in the control plants. The results confirm that peanut plants formed mycorrhizal relationship. This relationship may have influenced plant development and growth.

### Effects of inoculation with *Funneliformis. mosseae* on peanut canopy structure

Canopy structure refers to the relative position and number of stems and leaves above the ground. There are two types of indicators, namely non-complex traits and complex traits. The former are the features extracted from the whole plant such as height, width, volume, and rough leaf area estimates, and the latter describe traits at the organ level such as accurate leaf area, leaf inclination, tiller angle, and fruit count^17^.

#### Non-complex traits of canopy structure

**Figure 1:**
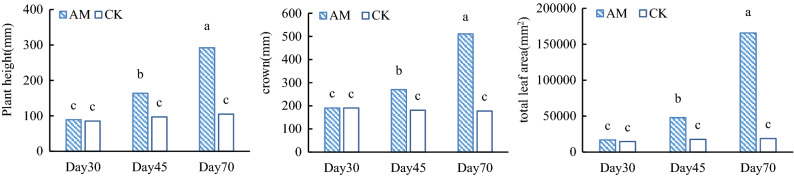
Effects of AMF inoculation on non-complex characteristics of the peanut canopy. Within each figure, columns without the same letter are significantly different by LSD (*p* < 0.05); AM, mycorrhizal (inoculated with the AMF); CK, uninoculated control; n = 8.

At 30 days the growth rates of mycorrhizal and uninoculated control plants were similar. At 45 days the growth rate of the mycorrhizal plants was significantly higher than that of the controls. The mycorrhizal plants continued to grow after 70 days. In contrast, the plant height of the control treatment increased but the leaves withered due to severe nutrient deficiency. The height, canopy width and total leaf area of mycorrhizal plants were 68.7, 49.7 and 71.1% greater than those of the controls after 45 days of growth and 179, 187 and 1020% greater at 70 days. AMF promoted plant growth and increased the total leaf area, and provided a prerequisite for increasing photosynthetic accumulation and further promoting plant growth.(Fig. 1).

#### Complex traits of canopy structure

**Figure 2:**
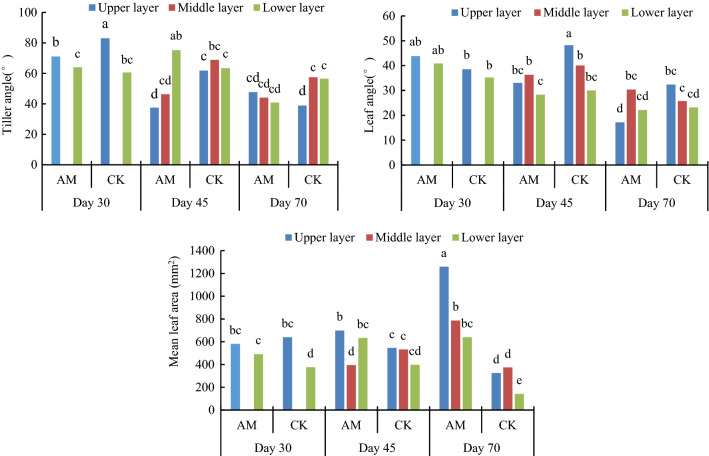
Effects of AMF inoculation on complex characteristics of the peanut canopy; within each parameter, bars without the same letter are significantly different by LSD (*p* < 0.05).

Figure 2 shows the effects of AMF inoculation on the composite characteristics of the peanut canopy. All indicator data in the figure were analyzed using one-way analysis of variance. At 30 days the new tillers were allocated to an upper layer and the old to a lower layer according to their growth order. At 45 days and 70 days the tillers were divided into upper, middle and lower layers as the tillers continued to increase (Fig. 2). At 30 days of growth the inoculated and control plants showed similar height, crown breadth and leaf area but subtle differences occurred in tiller angle and leaf angle with the inoculated plants showing a significantly smaller tillering angle and a larger leaf angle than the controls and a more pronounced decrease in average leaf area than in the controls, suggesting that the mycorrhizal plants were more upright (Fig. 2).

After 45 days of growth (Fig. 2) the tiller angle of the upper, middle, and lower layers in the mycorrhizal plants decreased gradually in the sequence: upper, middle and lower but did not differ significantly in the controls. In addition, the tiller angle in the upper and middle layers of the mycorrhizal treatment was significantly smaller than in the control plants but the lower part was larger than in the controls. By contrast, the control plants were poorly layered. Because the newly developed tillers had smaller tiller angle the above results indicate that there were more fresh tillers in the mycorrhizal treatment than in the controls after 45 days of growth, suggesting more rapid growth of the mycorrhizal plants than of the controls. Moreover, the leaf inclination angles in the upper, middle and lower layers of the mycorrhizal plants were smaller than those in the controls, indicating that the leaves of the mycorrhizal plants were relatively flat, providing a larger area to receive solar radiation and conducive to photosynthesis. The average leaf area of the mycorrhizal plants was larger in the upper and lower layers than in the middle layer. This structure may reduce the shading of the lower leaves and increase light transmittance for photosynthesis. There was no difference in the average leaf area among different canopy layers in the controls (Fig. 2).

**Figure 3:**
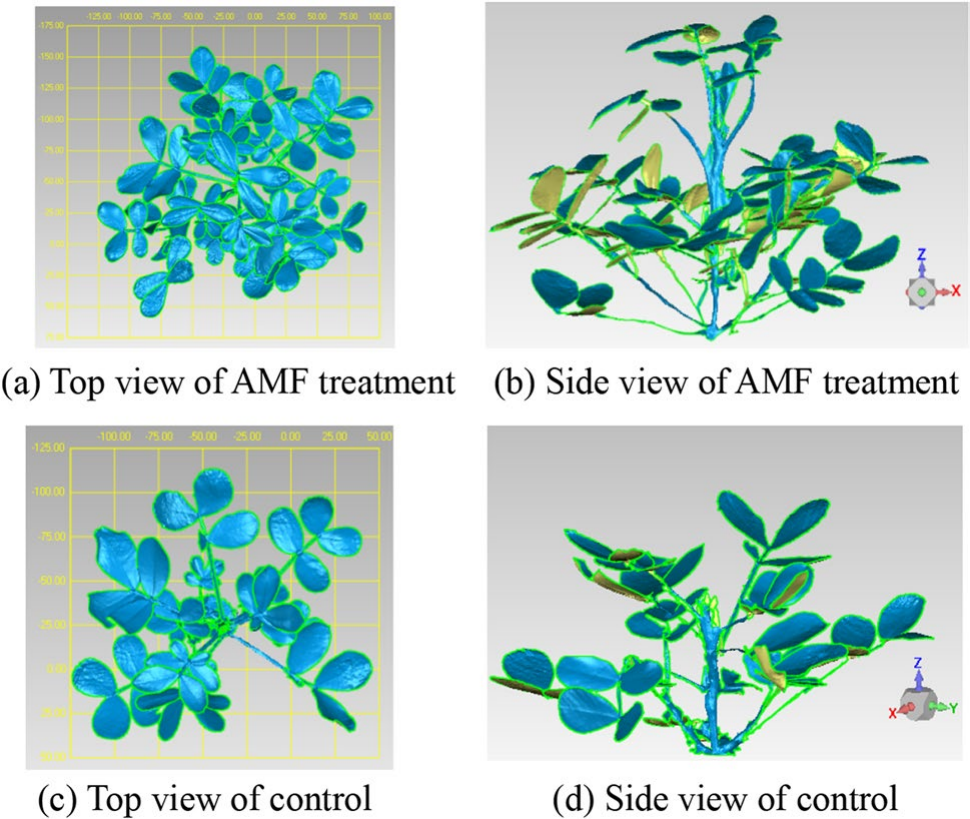
Top view and side view of the peanut canopy in different treatments after 45 days of growth.

After 70 days of growth (Fig. 2) the tiller angle in the AMF treatment decreased gradually from the upper to the lower layer and that of the control was smaller in the upper layer, indicating that the control plants were still growing and the mycorrhizal plants were already at maturity and their growth had slowed down or stopped. In addition, the inclination angle of the upper leaves in the mycorrhizal treatment was significantly smaller than those of the middle and lower layers because the upper leaves were larger and unable to stand upright and appeared bent and sagging. The configuration of the shoots of the mycorrhizal plants formed an angle gradient from top to bottom, indicating that the upper layer inclination angle was the minimum and that of the lower layer was the maximum. The upper and lower leaves would not shade each other, thereby increasing the foliar area exposed. The total leaf area was also larger in mycorrhizal than in control plants at the same layer and the same time periods. The results are consistent with the changes in leaf inclination and tiller angle. Overall, inoculation with *F. mosseae* promoted a more functional and hierarchical canopy structure so as to provide a better structure for photosynthesis (Fig. 3). The average leaf area of the inoculated treatment increased with time and nutrient deficiency in the intermediate and later growth stages of the uninoculated controls led to smaller newly-formed leaves and dead and yellow older leaves. Thus, average leaf area tended to decline.

#### Effects of AMF inoculation on leaf area index

Leaf area index is the ratio of the total leaf area to the projected area. Although the total area of the inoculated leaves increased rapidly during growth the canopy expansibility and the projected area also increased, thus the leaf area index increased slightly. After 30 days of growth the leaf area index was not significantly different between mycorrhizal plants and controls. After 45 and 70 days of growth the leaf area index of mycorrhizal plants was significantly higher than that of the controls, indicating that AMF colonization increased the leaf area index.

After 30 days of growth (Table 1) the plants were short and their leaf area index was small. There was little difference between inoculated and control plants. Leaf area index gradually increased after 45 days and showed significant difference between mycorrhizal and control plants. The plants later entered the mature stage. Their lower layer leaves and sheltered leaves gradually withered and their leaf area index began to decline.

**Table 1:** Effects of mycorrhizal soil inoculation on leaf area index of peanut.

Time	Day 30		Day 45		Day 70	
Treatment	Mycorrhizal	Control	Mycorrhizal	Control	Mycorrhizal	Control
Leaf area index	1.59b	1.59b	1.91a	1.77ab	1.78ab	1.70ab

Mean values not followed by the same letter are significantly different by least significant difference (LSD, *p* < 0.05).

### Effects of AMF inoculation on photosynthesis

#### Net photosynthetic rate

Table 2 shows the effects of AMF inoculation on net photosynthetic rate. The values of the same index at different times and of different treatments and different levels have been collated to allow unified analysis and overall comparison. The plants were short and their canopy was divided into two layers according to the tillering time after 30 days of growth. At this time the differences in net photosynthetic rate between different treatments and different layers were not significant. After 45 and 70 days of growth the net photosynthetic rate showed a tendency toward upper layer ≥ middle layer > lower layer. The results indicate that leaf structure plays an important role in leaf net photosynthetic rate. There was no difference in net photosynthetic rate between different layers in the controls (Table 2), likely due to the slow growth and short stature of the controls and to the lack of significant difference between layers. In addition, the leaf area index was low and the leaves in the middle and lower layers were blocked and had similar light reception.

**Table 2:** Net photosynthetic rate of peanut under different treatments at different canopy layers and growth periods in μmol m^−2^ s^−1^.

Period layer	Growth					
	Day 30		Day 45		Day 70	
	Mycorrhizal	Control	Mycorrhizal	Control	Mycorrhizal	Control
Upper	7.25c	6.17c	15.97a	5.74c	12.77b	7.83c
Middle	nd	nd	15.41a	4.39c	10.54b	4.92c
Lower	6.09bc	4.66bc	10.85a	3.58c	7.57b	5.84bc

Within each row, mean values not followed by the same letter are significantly different by LSD (*p* < 0.05); nd, not determined.

After growth for 45 days and 70 days (Table 2) the net photosynthetic rate at different positions was significantly higher in the mycorrhizal plants than in the controls. Mycorrhizal inoculation increased the net photosynthetic rate of the upper, middle and lower leaves by 345, 251 and 204%, respectively, indicating that inoculation increased the net photosynthetic rates of individual leaves. After growth for 45 days the canopy growth of mycorrhizal plants reached a maximum. Then the plants started to enter the fruit filling stage. During this stage the leaves began to age and the photosynthetic rate decreased. By contrast, control plants grew slowly and their canopy continued to grow slowly until later growth stages, with photosynthesis still increasing. Therefore, the difference in photosynthetic rate between inoculated plants and controls reached a maximum at intermediate growth stages.

The utilization of light energy and the level of photosynthetic characteristics determine the pod fullness in later growth stages^18^. AMF inoculation increased the net photosynthetic rate and also the leaf area for photosynthesis, thereby greatly increasing total accumulation of photosynthate, and this is very important in increasing peanut yield.

#### Effects of AMF inoculation on other photosynthetic indices

Table 3 shows that after 45 days of growth, the net photosynthetic rate, stomatal conductance and transpiration rate of the inoculation treatment were all higher than that of the control, while WUE was lower than that of the control. Therefore, the inoculation treatment promoted the absorption of water and nutrients by the peanut root system, increased its photosynthetic rate, and made the plant cells full of water to meet the growth needs.

**Table 3:** Other peanut photosynthetic indicators under the different treatments for 45 days.

Canopy layer indicator	Upper		Middle		Lower	
	Mycorrhizal	Control	Mycorrhizal	Control	Mycorrhizal	Control
Stomatal conductance(mol·m^−2^·s^−1^)	0.27a	0.07c	0.28a	0.05c	0.2b	0.04c
Intercellular CO_2_ concentration (μmol/mol)	269b	320a	278b	245c	280b	244c
Transpiration rate (mmol·m^−2^·s^−1^)	9.77a	1.95c	10.0a	2.39c	7.53b	1.96c
Water use efficiency (g/kg)	1.68c	2.92a	1.55c	1.82b	1.49c	1.84b

Within each row, mean values not followed by the same letter are significantly different by LSD (*p* < 0.05).

### Correlation analysis between canopy structure and photosynthetic characteristics

In the middle growth stages more than two-thirds of the canopy layer is the main light-absorbing layer. Correlation analysis of the canopy structure and its upper structure parameters with photosynthetic characteristics at 30, 45 and 70 days of growth (Table 4) shows that plant height, crown width and total leaf area were positively correlated with net photosynthetic rate, intercellular CO_2_ concentration and stomatal conductance, indicating that the more developed the canopy the stronger the photosynthetic ability. By contrast, leaf inclination and tiller angle were significantly negatively related to net photosynthetic rate, intercellular CO_2_ concentration, and stomatal conductance, indicating that the more horizontal the leaves were, the larger the leaf area receiving light, the more upright the tillers, and the stronger the photosynthetic activity, and so the higher the rate of photosynthesis. Moreover, there was no significant correlation between average leaf area and photosynthesis.

**Table 4:** Correlations between canopy structure and photosynthetic characteristics of peanut.

Photosynthetic characteristic index	Canopy structure indexes						
	Plant height	Crown size	Total blade area	Leaf area index	Leaf angle	Mean blade area	Transpiration rate
Net photosynthetic rate	0.495**	0.524**	0.492**	0.326*	-0.350*	0.363	-0.601**
Intercellular CO_2_ concentration	0.396**	0.346*	0.365*	0.223	-0.124	0.139	-0.419*
Stomatal conductance	0.616**	0.587**	0.594**	0.338*	-0.364*	0.319	-0.671**
Transpiration rate	0.382**	0.327*	0.312*	0.321*	-0.352*	0.153	-0.611**

*, *p* = 0.05; **, *p* = 0.01 by two-tailed Student’s t-test, n = 48.

The canopy is the only site of photosynthesis. AMF inoculation increased the height, crown width, total leaf area, average leaf area and leaf area index, and reduced leaf inclination and tiller angle. Correlation analysis shows that the height, crown width, and total leaf area were significantly positively related to net photosynthetic rate, intercellular CO_2_ concentration, and stomatal conductance, while leaf inclination and tiller angles were negatively related to net photosynthetic rate, intracellular CO_2_ concentration, and stomatal conductance, indicating that changes in canopy structure due to AMF altered the microenvironment of the canopy to some extent, and this indirectly affected its ability to conduct photosynthesis.

### Effects of AMF inoculation on plant nutrient uptake and biomass accumulation

#### Plant nutrient contents

The contents of total nitrogen, total phosphorus and total potassium in the leaves, stems and roots of the inoculated plants were significantly higher than those of the controls (Table 5). These results are similar to those of Qiu et al.^16^ and indicate that AMF promoted plant uptake of nutrients, thus promoting plant growth.

**Table 5:** Nutrient accumulation in the roots, stems and leaves of peanut.

Measurement	Nitrogen (mg)			Phosphorus (mg)			Potassium (mg)	
Treatment Plant part	Mycorrhizal	Control		Mycorrhizal		Control	Mycorrhizal	Control
Leaves	112a	7.07b		2.58a		0.05b	5.75a	0.41 ± 0.18b
Stems	70.4a	30.1b		1.38a		0.35b	4.72a	1.13 ± 0.22b
Roots	22.4a	20.1a		0.44a		0.28a	0.73a	1.1 ± 0.55a

Nutrients accumulated in each plant part (mg) = concentration of nutrient in each plant part (mg kg^-1^) × dry weight of the plant part (g) × 10^–3^; within each nutrient, mean values not followed by the same letter are significantly different by LSD (*p* < 0.05).

#### Effect of AMF inoculation on biomass accumulation

*F. mosseae* inoculation significantly increased dry biomass matter accumulation and plant yield and greatly promoted plant growth (*p* < 0.05). The results are similar to those of Xiao et al.^19^ and in line with the changes in canopy structure. The canopy structure of mycorrhizal plants become more developed than that of the controls. The leaf angles of the upper and lower layers adjusted to receive more light and increased the photosynthetic rate to increase plant yield (Table 6). The control group due to lack of sufficient nutrients, the leaves began to wither and fall at the later stage, which significantly reduced the aboveground biomass, and the fruit was small or even non-existent.

**Table 6:** Effects of AMF inoculation on dry matter accumulation and yield of peanut.

Treatment	Above-ground (g)	Roots (g)	Yield (g)
Mycorrhizal	15.60a	1.39a	6.49a
Control	1.90b	0.94b	0.02b

Within each column, mean values not followed by the same letter are significantly different by LSD (*p* < 0.05).

## Discussion

Canopy structure directly affects the efficiency of photosynthesis. Canopy structural characteristics of crops have a significant impact on the ability of the canopy to intercept photosynthetically active radiation^20^ and also on the microenvironment within the canopy such as temperature, humidity and light intensity^3^, and this affects crop photosynthesis and yield.

Leaf area index reflects the status of plant growth and foliar utilization of light energy^21^. When the index is < 5.5 the larger the leaf area index the higher the light reception and the greater the utilization of light energy^22^. We found the maximum value of the leaf area index to be ˂ 5.5 in both mycorrhizal and control plants. In the intermediate and later growth stages the leaf area index of mycorrhizal plants was greater than that of controls, indicating that AMF inoculation increased the effective radiation utilization by the canopy, similar to the findings of Wang et al.^21^. Here, after inoculation the tiller angle of the upper layer was higher than that of the middle layer, and that of the middle layer was higher than that of the lower layer. In addition, the tiller angles of the upper and middle layers of mycorrhizal plants were significantly lower than those of the controls, and the tiller angle of the lower layer was significantly higher than that of the controls. This may be related to the lateral branches in the lower layer growing along the ground and those in the middle and upper layers growing upright^23,24^, indicating an enhanced configuration of mycorrhizal plants.

The optimum temperature range of the peanut canopy during the pod-in stage is 23–28 °C and within this range the higher the temperature the more the pods are produced. The optimum range of relative humidity at the pod-in stage is 70–80%^25^. If the canopy is too dense the light energy utilization rate will decline, the ventilation and air permeability of the canopy will be poor, water evaporation will be slow, and the relative humidity will be too high, all of which are not conducive to plant growth. If the canopy is too sparse the leaves receive too much light, the leaf water evaporation is high, and the relative humidity of the canopy is low, all of which are also not conducive to growth. When the critical value of the leaf area index of 5.5 is not exceeded, AMF inoculation increases the number of leaves, leading to increased canopy density to a certain extent and making the canopy temperature and humidity more conducive to plant growth.

AMF make the plant configuration more well developed and promote the maximization of photosynthesis. It may be that AMF first affect the root function^26^, promoting nutrient uptake and transporting more nutrients to the aboveground parts to support the stems, increase leaf robustness and expand the canopy. Chen and Han^27^ found that with sufficient nutrients the canopy structure of *Glycine max* was loose, conducive to the formation of yield factors. By contrast, in nutrient-poor conditions the canopy was blocked, limiting the formation of yield factors. AMF inoculation can effectively increase nutrient uptake^26^, ensure adequate nutrition of plant aboveground parts and optimize canopy structure to increase the efficiency of photosynthesis. The aboveground parts of mycorrhizal plants can transfer more carbohydrates to the roots, and the growth and development of mycelia also require more carbohydrate for maintenance of growth. AMF can promote plant uptake of N, P, K and other mineral nutrients from the soil by peanut^28^, thereby directly or indirectly increasing photosynthesis.

Water use efficiency (WUE) is the ratio of net photosynthetic rate to transpiration rate and is an important index of the drought resistance of plants. When water resources are scarce, WUE is an important way to coordinate the contradiction between plant productivity and water consumption^29^. Stomatal conductance is a common route for CO_2_ and water vapor to enter and exit, but theoretically the diffusion resistance of CO_2_ is lower than that of water vapor, and the influence of stomatal conductance is greater on photosynthetic rate than on transpiration rate. Studies show that WUE increases with decreasing stomatal conductance^30^. Here, the control plants had a weak water and nutrient uptake capacity and a low intracellular water content. Stomatal conductance was reduced to meet the growth needs, resulting in a higher water use efficiency of the controls than of the inoculation plants. AMF inoculated peanut reached the maximum net photosynthetic rate after 45 days of growth and began to complete the transition from vegetative to reproductive growth. By contrast, control plants were still at the slow growth stages until 70 days. It is possible that the root system of inoculated plants could take up more nutrients and transfer them to the aboveground parts. AMF inoculation can improve canopy structure to make more effective use of solar energy as discussed above. AMF are not photosynthetic and cannot break down complex organic molecules in the soil as a source of carbohydrates for growth and are entirely dependent on the carbon supply from the host plants^10,10^. Mycelia can utilize 4–20% of the photosynthetic products of the host plants^32^, thus increasing the demand of the host plants for carbon. Increased carbon demand by plants will further accelerate the tricarboxylic acid cycle in the photosynthetic reaction, regeneration of RuBP^33^, transport of photosynthetic products to the aboveground parts, photosynthetic reaction processes, and plant photosynthetic rate. The utilization of photosynthetic products including sucrose and glucose by AMF promotes the transportation of sucrose from leaves to roots, thereby increasing photosynthesis. Wu et al.^34^ inoculated *Citrus reticulata* seedlings with AMF and found that sucrose and glucose contents in *C. reticulata* leaves were significantly positively correlated with AMF colonization rate. The association between AMF and host plants can speed up the transport of photosynthetic products from leaves to roots, thereby reducing the concentration of carbohydrates in the aboveground parts of plants, stimulating photosynthesis to meet plant growth needs, and increasing CO_2_ fixation. This may be because AMF promote the transport of tricarbonose in plants, accelerate the Pi cycle, and further accelerate the tricarboxylic acid cycle, thus increasing plant photosynthetic ability^35^. AMF can improve plant nutrition status, carbon fixation^36^, organic matter synthesis and accumulation, as well as carbon transport distribution, fixation and cycling in the ecosystem.

## Conclusions

The AMF colonized the peanut roots and may have formed a potentially symbiotic association. AMF inoculation increased nutrient uptake, plant height, crown width, total leaf area, dry matter accumulation and yield. The tiller angle of inoculated plants showed the trend: upper layer < middle layer < lower layer, modifying the canopy structure to a more hierarchical configuration, and there was no significant effect among different layers in the uninoculated controls. AMF inoculateion changed the structure of the canopy and further affected photosynthesis. AMF inoculation increased the net photosynthetic rate of leaves in different layers, and the net photosynthetic rate was significantly positively correlated with leaf height, crown width, total leaf area and leaf area index, and significantly negatively correlated with leaf upper dip angle and tiller angle. The results indicate that the use of AMF may have some potential in practical agriculture. This study has concentrated on canopy structure but has not explored the effects of AMF on root traits, a topic that merits future investigation.

## Materials and methods

### Ethics statement

The authors of the above articles promise to: all plant studies were carried out in accordance with relevant institutional, national or international guideline in the methods section.

### Experimental materials

A strain of the AMF *Funneliformis mosseae* provided by the Laboratory of Microbial Reclamation, China University of Mining and Technology (Beijing), was used here. The mycelial length was 3.12 m g^-1^ and the spore density 26 g^−1^ substrate. The peanut variety used was *Arachis hypogaea* cv. The Variety was Fuyu Siguanhong and the Registration number was GDP Peanut (2018) 220,156, Silihong purchased from China Seed Trading Network. A high temperature-sterilized river sand was selected as the experimental soil to explore the effects of AM fungal inoculation on peanut growth in nutrient-poor conditions. The river sand hads content of available phosphorus and available potassium of 1.25 and 41.3 mg kg^−1^, respectively, and the organic matter content was 2.71 g kg^−1^.

### Experimental design

The experiment was conducted outdoors at the Microbial Reclamation Laboratory, China University of Mining and Technology (Beijing). In detail, a total of 16 pots were randomly assigned into AMF-inoculated and uninoculated control treatments with eight replicate pots each. Each inoculated pot contained a mixture of 5 kg river sand and 50 g of AMF inoculum composed of AMF root segments and rhizosphere soil. Each control pot contained a mixture of 5 kg river sand and 50 g of sterilized AMF inoculum. Five peanut seeds were sown in each pot on 25 May 2019. At the true leaf stage only one seedling was retained in each pot and the pots were watered to maintain soil water content at 70–80% of the water holding capacity (WHC) based on weight.

In addition, each pot was supplied with nutrient solution containing NH_4_NO_3_, KH_2_PO_4_, and KNO_3_ to maintain the contents of N, P, and K per kilogram of soil at 100, 25, and 150 mg, respectively. The plants were observed after growth for 30, 45 and 70 days and harvested on day 70. The above- and below-ground dry weights of peanut were determined using the oven dry method. In addition, 0.1 g of the ground, dried roots, stems and leaves were digested in a mixture of sulfuric acid and hydrogen peroxide. A portion of each sample was used to determine the total nitrogen content using a Kjeldahl instrument, and the remainder was used to determine total nitrogen content using an ICP-OES spectrophotometer. Some fresh fine root samples were collected and used for determination of total phosphorus and total potassium^37^. A small amount of fresh fine root sample was randomly collected. After immersion in 10% KOH (w/w) for 24 h and rinsing with water they were stained using acid fuchsin:lactic glycerol solution as reported previously. Fifteen root sections were randomly selected, prepared on slices, and observed under an optical microscope to determine the percentage of root length colonized^38^.

### Measurement of plant three-dimensional structures

#### General procedures

The point cloud data of the three-dimensional canopy structure of peanut were obtained using a portable HandySCAN 700 three-dimensional laser scanner (https://www.creaform3d.com.cn/zh/ji-liang-jie-jue-fang/bian-xi-shi-3d-sao-miao-yi-goscan-3d) with a scanning accuracy of 0.5 mm after 30, 45, and 70 days of growth in an indoor windless environment. After the scanning was completed the grid data were created and exported in the STL format. The data were then processed using VXelements(v.2.0, https://www.creaform3d.com/en/node/9307/attachment/newest) with the main steps of coordinate transformation, noise removal, model repair and data extraction to obtain an independent and complete three-dimensional peanut model^39^.

#### Extraction of data

**Figure 4:**
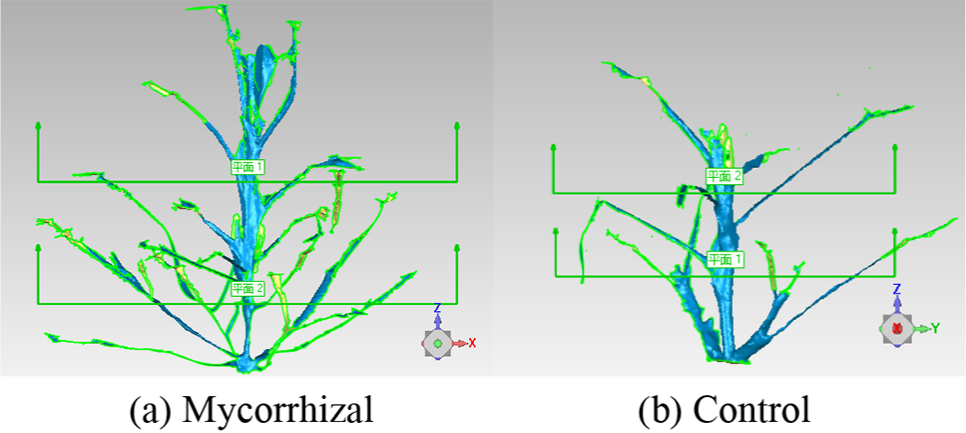
Stratification of stem structure in the peanut canopy.

The plant height (cylinder height) and crown width (cylinder diameter) were extracted using the cylinder in the "Feature" tool as the smallest cylinder surrounding the plant^17^. The total leaf area and average leaf area of different layers were calculated using the Selection and Measurement tools^39^. The planes where peanut leaves were located were obtained using the best fit method and the angle between the plane and the ground, that is, the leaf inclination angle, was obtained using the measurement tool and the trigonometric function method^40^. The angle between the tiller and upright direction, that is, the tiller angle, was calculated using the Measurement tool and trigonometric function method. The projected area was calculated using the supervised classification method in the grid background of the screenshot view. The ratio of the total leaf area to the projected area was used to calculate the leaf area index (LAI)^41^. In the intermediate and later growth stages the canopy was divided into upper, middle and lower layers according to tiller height and leaf age (Fig. 4).

Peanut reached peak photosynthesis at 10:00–13:00 on a day with sunny and stable weather. The stomatal conductance, intercellular CO_2_ concentration, transpiration rate and net photosynthetic rate of peanut leaves at different layers were therefore measured on a clear and cloudless day using an LI-6400 XT portable CO_2_/H_2_O analysis system (Li-COR Inc., Lincoln, NE) . The effective radiation of the light source was PAR 1000 m^2^ mol/(m^2^ s), the leaf chamber used was 2 × 3 cm, and the gas flow rate was 500 mmol s^-1^. Three groups of leaves were determined randomly at each layer of each plant.

The yield of each peanut plant was determined and then the average yield of mycorrhizal and control peanut was obtained.

### Data processing

Microsoft Excel 2010 was used for data collation and PowerPoint 2019 was used for drawing the Figures. The SPSS(v.20.0; IBM, Armonk, NY, USA)was used for one-way analysis of variance (ANOVA) and correlation analysis and the significance level was P < 0.05.

### Significance statement

The AMF and peanut formed a potentially mutualistic relationship. The AMF increased plant growth and made the plant canopy structure more hierarchical than the control. The changes in canopy structure of mycorrhizal plants are attributable to increased leaf area, chlorophyll content and photosynthetic rate and to plant nutrient uptake, leading to higher peanut biomass.

There was a reciprocal relationship between the AMF and the plants that promoted plant nutrient uptake and made the plant canopy well developed and layered. AMF increased net photosynthetic rate and photosynthetic leaf area, thereby increasing plant biomass and yield.

### Submit statement

The authors of the above articles promise to: all plant studies were carried out in accordance with relevant institutional, national or international guideline in the methods section.
